# User-centred assistive SystEm for arm Functions in neUromuscuLar subjects (USEFUL): a randomized controlled study

**DOI:** 10.1186/s12984-020-00794-z

**Published:** 2021-01-06

**Authors:** Valeria Longatelli, Alberto Antonietti, Emilia Biffi, Eleonora Diella, Maria Grazia D’Angelo, Mauro Rossini, Franco Molteni, Marco Bocciolone, Alessandra Pedrocchi, Marta Gandolla

**Affiliations:** 1 NeuroEngineering And medical Robotics Laboratory, NearLab, Department of Electronics, Information, and Bioengineering, Politecnico di Milano, Via Giuseppe Colombo 40, 20133 Milan, Italy; 2 Scientific Institute IRCCS E. Medea, Via Don Luigi Monza 20, 23842 Bosisio Parini, Italy; 3 Valduce Hospital, Villa Beretta Rehabilitation Center, Via Nazario Sauro 17, 23845 Costa Masnaga, Italy; 4 Department of Mechanical Engineering, Politecnico di Milano, Via Giuseppe La Masa 1, 20156 Milan, Italy

**Keywords:** Exoskeleton, assistive device, neuromuscular disorders, upper limb

## Abstract

**Background:**

Upper limb assistive devices can compensate for muscular weakness and empower the user in the execution of daily activities. Multiple devices have been recently proposed but there is still a lack in the scientific comparison of their efficacy.

**Methods:**

We conducted a cross-over multi-centric randomized controlled trial to assess the functional improvement at the upper limb level of two arms supports on 36 patients with muscular dystrophy. Participants tested a passive device (i.e., Wrex by Jaeco) and a semi-active solution for gravity compensation (i.e., Armon Ayura). We evaluated devices’ effectiveness with an externally-assessed scale (i.e., Performance of the Upper Limb-PUL-module), a self-perceived scale (i.e., Abilhand questionnaire), and a usability scale (i.e., System Usability Scale). Friedman’s test was used to assess significant functional gain for PUL module and Abilhand questionnaire. Moreover, PUL changes were compared by means of the Friedman’s test.

**Results:**

Most of the patients improved upper limb function with the use of arm supports (median PUL scores increase of 1–3 points). However, the effectiveness of each device was related to the level of residual ability of the end-user. Slightly impaired patients maintained the same independence without and with assistive devices, even if they reported reduced muscular fatigue for both devices. Moderately impaired patients enhanced their arm functionality with both devices, and they obtained higher improvements with the semi-active one (median PUL scores increase of 9 points). Finally, severely impaired subjects benefited only from the semi-active device (median PUL scores increase of 12 points). Inadequate strength was recognized as a barrier to passive devices. The usability, measured by the System Usability Scale, was evaluated by end-users “good” (70/100 points) for the passive, and “excellent” (80/100 points) for the semi-active device.

**Conclusions:**

This study demonstrated that assistive devices can improve the quality of life of people suffering from muscular dystrophy. The use of passive devices, despite being low cost and easy to use, shows limitations in the efficacy of the assistance to daily tasks, limiting the assistance to a predefined horizontal plane. The addition of one active degree of freedom improves efficacy and usability especially for medium to severe patients. Further investigations are needed to increase the evidence on the effect of arm supports on quality of life and diseases’ progression in subjects with degenerative disorders.

*Trial registration* clinicaltrials.gov, NCT03127241, Registered 25th April 2017. The clinical trial was also registered as a post-market study at the Italian Ministry of Health.

## Introduction

Muscular dystrophies (MDs) are characterized by progressive muscular weakness. Although the degree of decline and the severity of the conditions might differ, MDs are generally disabling in time. Most people with MDs eventually lose the ability to walk and the arm function is often impaired [1, 2]. With the current life expectancy, people with MD could live with impaired upper limbs’ function for a long period of time, either in DMD or in LGMD [3, 4]. If left unsupported, they may be seriously limited in activities of daily living (ADLs) and restricted in social participation for the same period of time [1, 5, 6]. If left unsupported, they may be seriously limited in ADLs and restricted in social participation for the same period of time [1]. Most of MDs affect proximal or shoulder muscles more than distal muscles. It results in impairments in motions that involve moving arms against gravity. As a consequence, this condition leads to losses in the range of motion (ROM) and functional movements [7]. The hand function, instead, could be partially preserved [8]. In this scenario, people with MDs can benefit from arm supports that compensate for the weight of their arms [9]. However, restoring a person’s ability to perform daily tasks with the upper limbs remains a difficult problem to overcome [10].

In recent decades, some efforts have been made in the field of upper limb assistive devices (ADs). These devices can be classified as passive, semi-active, or fully-active devices. Passive arm supports are used to reduce or eliminate the effect of gravity on a working plane. They use springs and elastic elements (e.g., Zonco Arm support by Zonco Arm) or counterweights (e.g., Sling by Focal Meditech), and allow the users to perform functional tasks with their weak residual muscle effort [11]. Otherwise, some passive devices are equipped with neither elastic elements nor with counterweights, they provide support only on the horizontal plane and assist the user during specific actions (e.g., Top by Focal Meditech). Semi-active devices, instead, are equipped with passive elements and an active one, that allows the end-user to adapt the level of antigravity support and to change the working plane. Finally, fully-active devices are complex mechatronic systems, where each degree of freedom is associated with an actuation system. In this way, they can provide extra support and augment the user’s residual capabilities [12]. The use of ADs to improve arm function was a key recommendation of a recent study on quality of life in people with Duchenne MD [13]. Lowering the gravity loading allows the user to employ the residual muscle force for movement, as well as for posture stabilization [10]. Some studies have been conducted to investigate the influence of ADs to restore the arm function in people with MD. They mainly involved passive or semi-active ADs. However, most of the studies included participants with MD together with patients with different neuromuscular disorders (e.g., amyotrophic lateral sclerosis, spinal muscular atrophy). The inclusion of patients with muscular disorders related to muscle fiber structure characterized by similar upper limb muscular and functional decline is missing. We found few studies that included only people with MD, focusing on Duchenne MD [14–16]. So far, the scientific community mainly focused on more diffused and more rapidly and severely evolving diseases (e.g., Duchenne MD, spinal muscular atrophy, amyotrophic lateral sclerosis), rather than slowly evolving diseases (e.g., Becker MD).

The effectiveness of ADs has been evaluated both in terms of objective and subjective measures. In general, patients reported improved confidence, dignity, and ability to engage in social situations, as well as increased independence in several activities thanks to ADs [7, 8]. Considering objective measures, ADs led to overall enhanced performance. Indeed, subjects obtained improvements in upper limb ROM [14, 17]. Additionally, the time required to complete ADLs, assessed through the Jebsen test of hand function, decreased [18]. Other studies evaluated ADs effect with subjective outcome measures. A pilot study [19] found improvements in patients’ perceived ability to perform ADLs and overall satisfaction with AD, evaluated with the Canadian Occupational Performance Measure. Gunn et al. [20] conducted an online survey on 55 patients with neuromuscular disorders, including MD. Results showed significant improvement in arm function for everyday tasks, according to patients’ opinions. According to a cross-sectional study [21], people with more limited functional abilities benefited most of arms support. In other studies [22] researchers asked patients to identify seven ADLs they considered important and that they wanted to see improved by using the AD. Also in this case, the AD had a positive effect on the ability of the users to perform important ADLs independently. Two studies, including one randomized control trial (RCT), have also reported that the use of AD for upper limb training may aid in slowing the loss of upper limb function in individuals with Duchenne MD [1, 23]. Nevertheless, we found only two studies [15, 16] that used a clinical scale specifically designed and validated to assess upper limb function for individuals with MD, i.e. the Performance of the Upper Limb (PUL) module [24]. Kooren and colleagues [15] evaluated a passive arm support prototype on four boys with Duchenne MD. Participants obtained an increase in the PUL module when wearing the prototype. In particular, upward and forward movements were easier to perform. A recent study [16] involved two commercial ADs: Armon Edero and Multilink with Elevation Assist. The authors used the PUL module and the Upper Limb Patient Reported Outcome Measure. Two MD patients reported that they were able to complete some ADLs faster, more independently, and with reduced compensatory movements. However, these last studies involved a very limited number of patients. Therefore, there is limited evidence supporting the effect of ADs on ADLs in individuals with MD.

A recent meta-analysis, performed by our group, summarized the current piece of evidence about the effect of wearable upper limb ADs for patients with neuromuscular diseases [25]. It demonstrated the effectiveness of arm supports to improve the ability to perform ADLs in people affected by degenerative neuromuscular disorders. The overall effect size was equal to 1.06 (95% CI 0.76 to 1.36). However, this work highlighted the main limitation of the available evidence in this field. Indeed, reported works are mainly pilot study on a restricted sample size of participants. Moreover, most of the studies involved people with several neuromuscular pathologies (e.g., MD, spinal muscular atrophy, arthrogryposis multiplex congenital), and do not focus on a specific pathology. Therefore, there is a need for high quality, well-designed, and large-scale studies. Moreover, according to this comprehensive work, rigorous studies should include both the subjective feedback given by patients themselves (i.e., self-perceived scales) and methodological scales performed by clinicians (i.e., externally-assessed scales). In this way, researchers and clinicians can obtain a more complete view of the device’s effects.

In this context, the USEFUL (User-centred assistive SystEm for arm Functions in neUromuscuLar subjects) project aims to field-test rigorously two commercial ADs for arm gravity compensation [26]. We conducted an RCT with crossover design on people diagnosed with MD. The goals of this project were (i) to assess the functional improvement at the upper limb level for MD patients induced by a passive and a semi-active device, and (ii) to compare the impact of these two different technologies on arm functionality.

## Materials and methods

### Participants

We recruited participants from in-patients and out-patients services at IRCCS E. Medea and Villa Beretta Rehabilitation Center. Eligible participants met the following inclusion criteria: (1) availability to sign the informed consent, (2) defined diagnosis of Duchenne MD, Becker MD, Limb-Girdle type 2 MD, or Congenital MD, (3) wheelchair dependence, (4) Muscular Rating Council (MRC) score [27] ranging from 1 to 4 for at least one muscle between deltoid and biceps brachii muscles, and (5) normal IQ level (evaluated through the Wechsler scale). We excluded patients if they had other major comorbidities, behavioral and psychiatric disturbances, or their family and caregiver did not comply with the study. All patients received detailed information about the study from the researcher or physician in charge at the center, and provided their written informed consent. The research protocol was approved by the Ethics Committee of both experimental centers (Comitato Etico Interaziendale delle province di Lecco, Como e Sondrio, protocol ID: 130/2016; Comitato Etico dell’IRCCS E. Medea Sezione Scientifica dell’Associazione La Nostra Famiglia, protocol ID: 013/16).

### Design

We conducted a cross-over multi-centric randomized controlled trial. The clinical trial was registered as a post-market study at the Italian Ministry of Health in November 2016. Moreover, it was registered on ClinicalTrials.gov (Identifier: NCT03127241) in April 2017. We aimed to on-field validate functional improvement induced by two upper limb ADs. All participants tested both devices and the starting device was randomly allocated. A computer-generated randomization sequence was made and an automated assignment system was used to ensure allocation concealment. Subjects were tested at three touchpoints: baseline assessment without arm support (i.e., T0), assessment after the use of the device A (i.e., T1 A), and assessment after the use of the device B (i.e., T1 B).

### Intervention

**Fig. 1:**
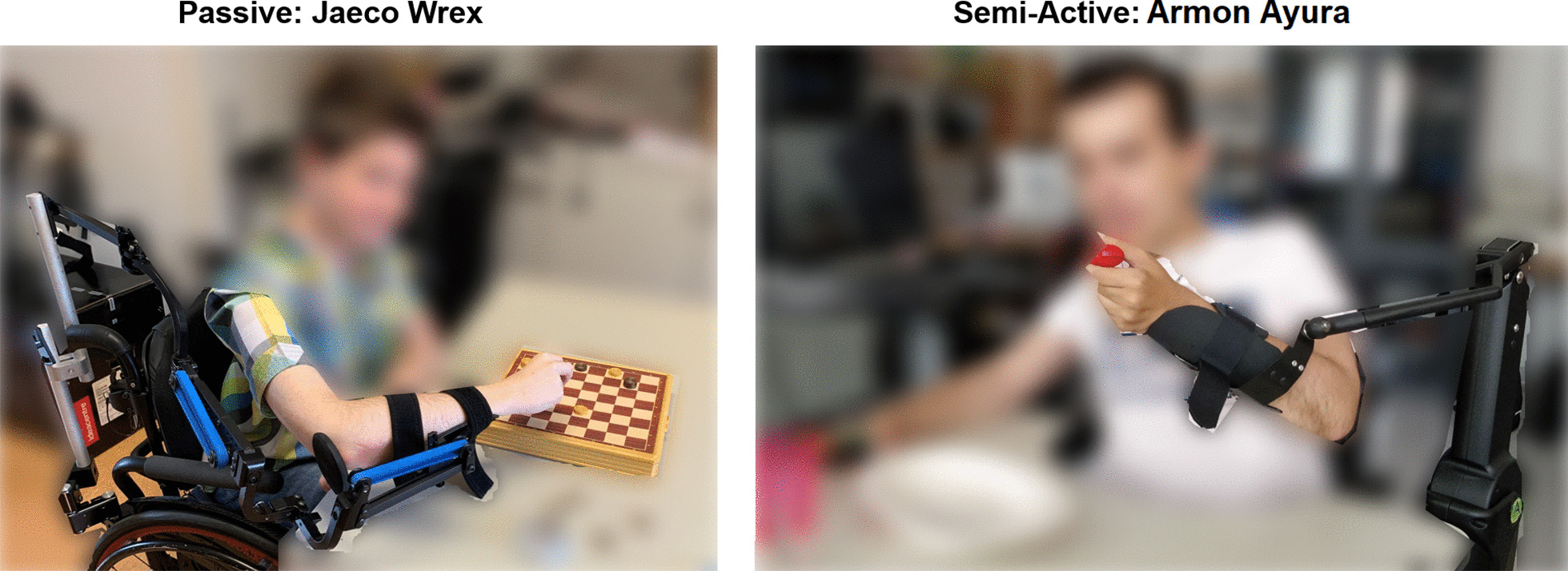
Commercial arm supports tested. **a** Wrex Jaeco and **b** Armon Ayura

The intervention involved the use of two commercial arms supports: Wrex (Jaeco) and Ayura (Armon) (Fig. 1). These two ADs models were chosen as representative of their respective category: passive and semi-active devices. Wrex is a passive body-powered antigravity exoskeleton. It is designed to enhance movement for individuals with neuromuscular disabilities of the upper extremities [28]. It uses linear elastic bands both for balance and to assist movements in three dimensions against the effects of gravity. The number of bands varies depending on the weight of the patient arm and his/her strength. The number of bands was chosen by the patient together with the physiotherapist during the assembly phase. In particular, the physiotherapist gradually increased the number of used bands. He/she stopped when the level of gravity compensation offered was optimized to perform both lifting tasks and activities at the table level. Then, participants were not able to change the number of bands. Ayura, instead, is a semi-active solution for gravity compensation. It is provided with buttons that allow the user (i) to modify the level of antigravity compensation, and (ii) to adjust the position of the arm relative to the trunk in the sagittal plane [26]. It can be electrically connected to the patient’s wheelchair if the patient is using a motorized wheelchair. Otherwise, it has to be connected to a standard domestic power line.

**Fig. 2:**
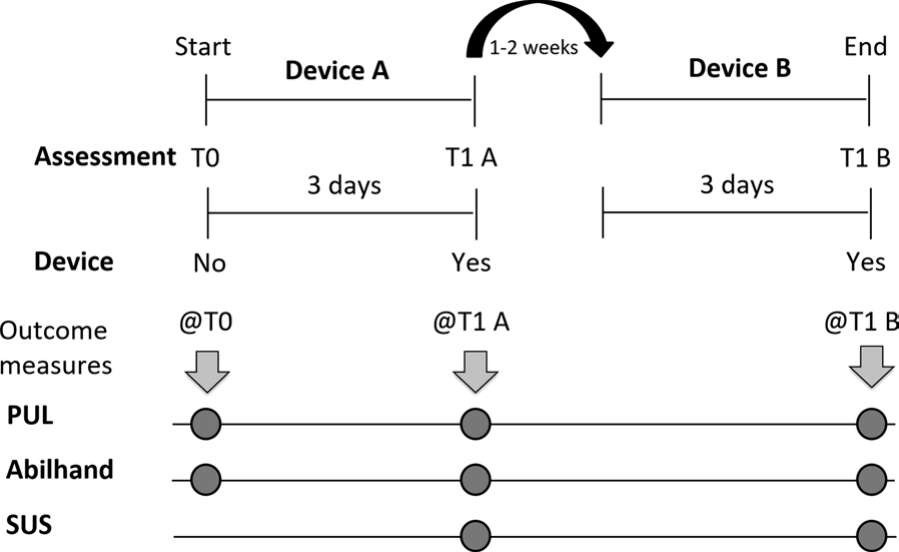
Intervention. T0: baseline assessment without arm support; T1 A: assessment after the use of the first device (Device A); T1 B: assessment after the use of the second device (Device B); *PUL* Performance of Upper Limbs module, *Abilhand* Abilhand scale, *SUS* System Usability Scale

The intervention is summarized in Fig. 2. After enrollment, participants underwent baseline assessment (@T0), including inclusion and exclusion criteria verification. Afterward, they were (randomly) assigned to the passive or semi-active device. A trained operator mounted the device on the patient’s wheelchair and customized it to enhance the patient’s comfort. Each device was mounted on the arm selected by the patient him/herself. Patients performed training with the device under the supervision of a physiotherapist for 2 to 4 h while performing ADLs. Participants were then encouraged to use the device during the following three days (intervention period), while executing ADLs at the table. In particular, they were instructed to wear the device for about 4 h per day. Typical activities included typing on a keyboard, using a tablet, drinking, eating, playing chess, etc. After the three days of training, the evaluation procedure was replicated for both devices. Before the evaluation, we asked participants if they used the device as recommended, and they confirmed. No differences have been reported in the use of the two devices. Given that a training effect was not foreseen for this pathology (neither observed), but that the decline of the pathology occurs over time, the two sections of the protocol were performed one after the other, controlling that the second phase of the protocol started with the same baseline conditions in terms of residual ability, as detected from PUL scale. The cross-over period was 1–2 weeks.

### Outcome measurements

Outcome measurements have been selected to evaluate upper limb functions in three domains.

(i) We evaluated externally-assessed functional condition with the PUL module (primary outcome measure)—version 1.2 [24]. It includes 22 items. The first one represents an entry item to define the starting functional level. The following 21 items are subdivided into shoulder level (4 items), elbow level (9 items), and wrist and fingers level (8 items). The shoulder-related level is administered only if the individual achieves a score of four or above on the entry item. Results are expressed in points over a 0-74 scale and higher scores represent higher assessed functional ability.
(ii) We assessed the self-perceived functional condition with the Abilhand questionnaire [29], where we asked participants to provide their perceived difficulty to perform 22 typical ADLs (e.g., open a door with a key, wash the hands). Possible answers to each item were “easy”, “difficult” or “impossible”. This questionnaire was analyzed following Vandervelde and colleagues’ guidelines [29]. Results are expressed in logit and higher scores represent higher perceived manual ability.
(iii) Finally, we investigated device usability with the System Usability Scale (SUS) [30]. It is a ten-items scale that provides a global view of subjective assessments of usefulness, as a combination of effectiveness, efficacy, and satisfaction of the system. The global scores were interpreted according to Bangor’s guidelines [31]. Results are expressed in points over a 0–100% scale and higher scores represent higher device usability.

At the end of all evaluations, we collected qualitative feedback. Indeed, we proposed to patients a final qualitative questionnaire to compare the two devices. We asked participants to choose which device they preferred to perform some daily tasks, together with questions about the perceived ease of use and the devices’ design. They could choose between the passive, the semi-active, both devices, and neither devices. Questions are listed in Table 3. In addition, we collected any verbal feedback patients felt to share with the researchers.

### Statistical analysis

Outcome measurements were collected at baseline (T0) and after the three-days training while wearing devices (T1). Data from each device were analyzed, irrespectively from the first device used by the participant. In the results section, therefore, we report the assessment scores divided into groups Semi-Active and Passive. To compute the sample size we considered as primary outcome measure the PUL module. Considering a Minimal Clinically Important Difference (MCID) of 12 points (suggested by clinicians), a within-subject standard deviation of 17.52 points [32], a statistical power of 80% and a significance level of 0.05, a sample size of 36 subjects was obtained. Given the non-normality of data, Friedman’s test was performed to assess possible significant functional gain using the assistive devices for the PUL module, and its 3 domains, and the Abilhand questionnaire. Post-hoc comparisons with Tukey’s Honestly Significant Difference correction were used to identify statistically significant differences between the three groups (i.e., T0, T1 Semi-Active, T1 Passive). PUL changes ($\Delta PUL$) with the two devices were compared by means of the Friedman’s test. Moreover, PUL changes were considered relevant if equal or higher than 4 points, according to the Minimum Detectable Change (MDC) of this scale [33]. We performed the same analyses stratifying patients into three groups according to the MRC index of the deltoid muscle. In fact, the aim of the study was to evaluate the functional improvements as induced by the use of two devices, which primarily have an antigravity effect at the shoulder level. Given that the primary muscle with antigravity function at shoulder level is the deltoid, we used the MRC scale at the deltoid level to stratify patients. In particular, we classified subjects as slightly impaired ($2.5 < MRC \le 4$), moderately impaired ($1 < MRC \le 2.5$), or severely impaired patients ($MRC \le 1$). All statistical analyses have been performed in MATLAB (version 2019a, RRID:SCR_001622), and data are presented as median [25th quartile-75th quartile]. A summary of all the statistical tests performed, sample sizes, and corrections for multiple comparisons (where applicable) is reported in the Additional File 1: Table S1.

## Results

### Participants

**Fig. 3:**
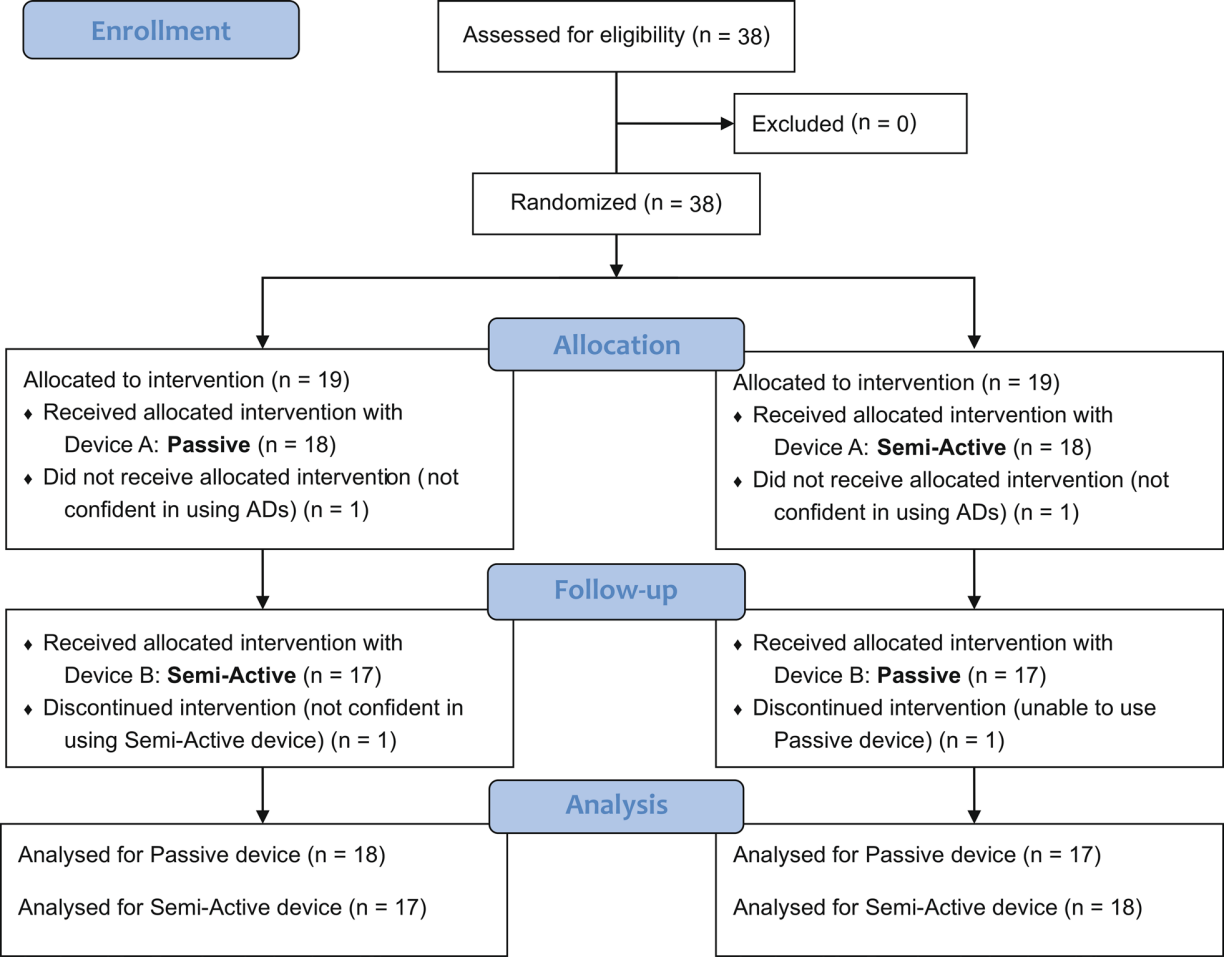
Participant CONSORT flow chart

Thirty-eight patients met the inclusion criteria and were recruited in the study from July 2017 to December 2019. Thirty-six completed the experimental protocol and were included in the analysis. Two participants left the study as they did not felt confident in the use of the devices. Indeed, one patient preferred to withdraw from the study because he/she suffered from generalized chronic joint pain and, after the first training day, he/she felt that the pain at the shoulder level was increasing. The other patient, instead, preferred to end the study because of his/her strong muscular contractures. 18 started with the passive and 18 with the semi-active device (Fig. 3). Table 1 outlines patients’ characteristics at baseline. One participant (ID29) was unable to use Wrex, because he/she felt not sufficiently supported given his/her level of residual ability. Another participant (ID4), instead, preferred not to use Ayura because of contractures at the elbow level that causes him not to feel confident with this type of end-effector device. Therefore, at the end of the study, we collected 36 measurements at T0, 35 at T1 Passive, and 35 at T1 Semi-Active.

**Table 1:** Participants’ baseline characteristics

Age, years	30 [19.5–54]
DMD	19 [15.5–21.5]
BMD	52 [38.5–58.5]
LGMD2	54 [31–57]
CMD	56 [51–61]
Gender, male/female	32/4
Pathology, DMD/BMD/LGMD2/CMD	16/8/10/2
Dominant arm, right/left	31/5
MRC deltoid	2.00 [0.5–3]
MRC biceps brachii	2.00 [1.5–2.75]

*DMD *Duchenne MD, *BMD* Becker MD, *LGMD2* Limb Girdle type 2 MD, *CMD* Congenital MD. Data are presented as median [25th quatrile–75th quartile]

**Fig. 4:**
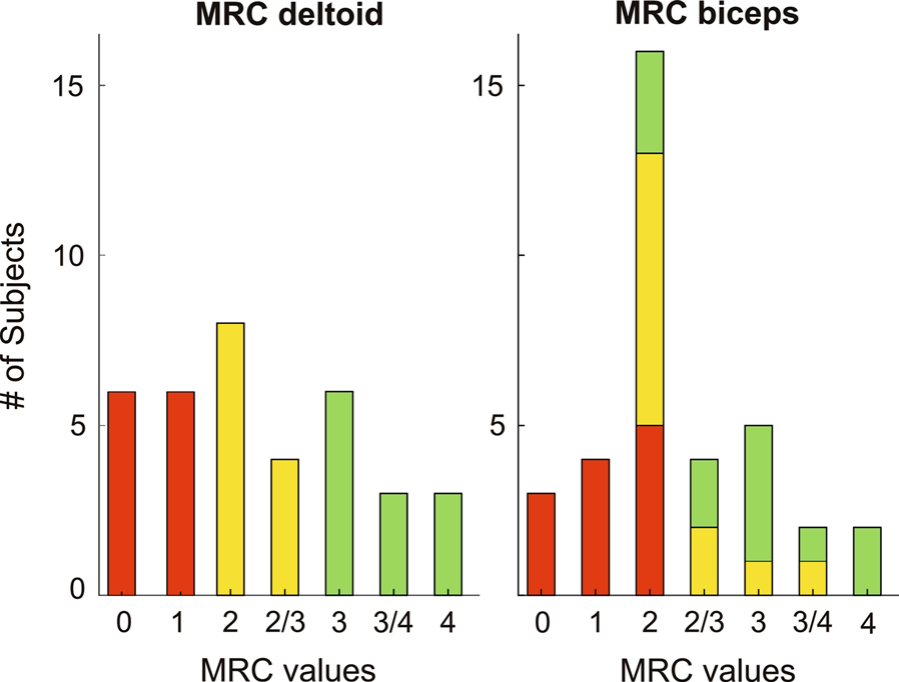
Frequency histogram of MRC values at deltoid and biceps muscles for all participants. Green: slightly impaired subjects; yellow: moderately impaired subjects; red: severely impaired subjects

As for the three sub-groups of patients (i.e., slightly, moderately, and severely impaired), they included 12 subjects each (Fig. 4). Slightly impaired patients were characterized by median [25th quartile–75th quartile] MRC of deltoid equal to 3.25 [3.00–3.75] and median MRC of biceps brachii of 3.00 [2.25–3.25], and they included 2 patients with DMD, 6 with BMD, 2 with LGMD2, and 2 with CMD. Six patients started with each device. In the moderately impaired group, the median MRC of deltoid was 2.00 [2.00–2.50], and the median MRC of biceps brachii was equal to 2.00 [2.00– 2.50]. The passive device was tested by five patients as the first device, whereas seven patients started with the semi-active one. This group included 7 participants with DMD, 1 with BMD, and 4 with LGMD2. Finally, the group of severely impaired patients was characterized by median MRC of deltoid equal to 0.50 [0.00–1.00] and median MRC of biceps brachii equal to 1.00 [0.50–2.00]. Seven patients started the trial with the passive and five with the semi-active device. Also in this group, there were 7 patients with DMD, 1 with BMD, and 4 with LGMD2. Full details of participants are reported in Table 1.

### Performance of the upper limb module results

**Fig. 5:**
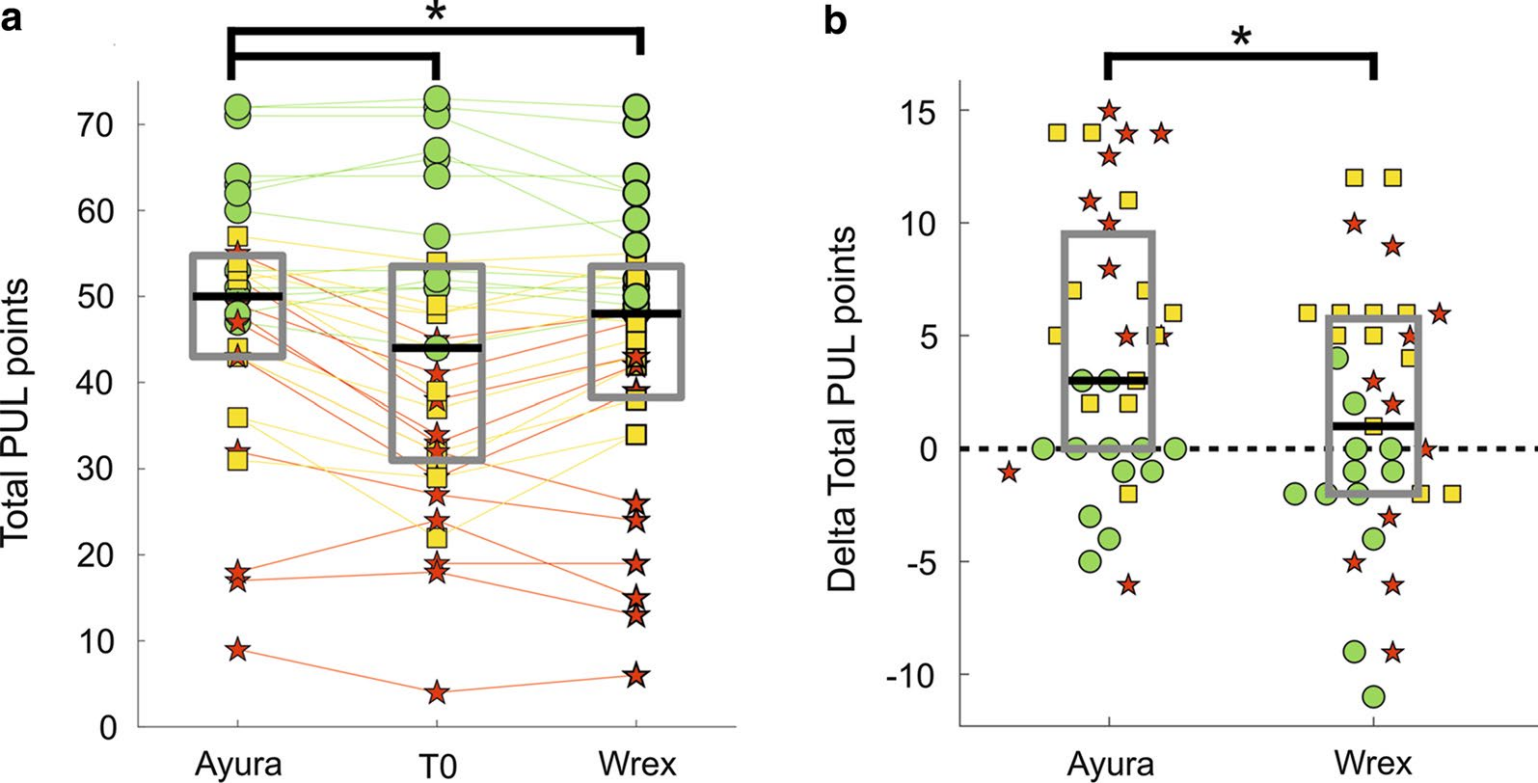
PUL module results. **a** Reports raw total PUL values (on a scale 0–74 points) at T0, T1 with the semi-active device, and T1 with the passive one. Green circles, yellow squares, and red stars represent PUL scores of slightly impaired, moderately impaired, and severely impaired subjects respectively. Straight lines connect data from the same participant. Grey boxes identify interquartile ranges and the black lines highlight the median values. Asterisks indicate statistical differences ($p-value < 0.05$) between groups, tested with Friedman’s and post hoc comparisons. **b** Reports Delta PUL values (i.e., the difference between T1, with the semi-active or passive device, and T0 PUL scores. Each data point is represented with the same marker and color code of **a**. The dashed black line indicates the zero delta value. Asterisk indicates a statistical difference between the two groups, as computed by means of Friedman’s test (p = 0.001015)

**Fig. 6:**
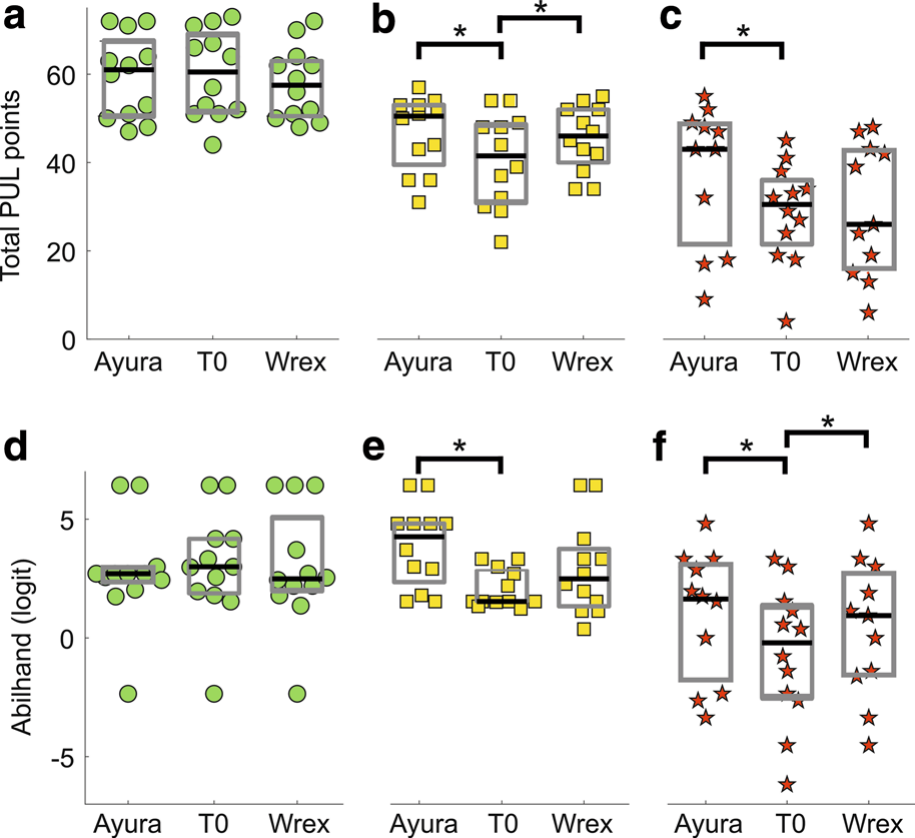
Upper panels: PUL module results for the sub-groups analysis. **a** slightly impaired subjects; **b** moderately impaired subjects; **c** severely impaired subjects. Lower panels: Abilhand scores, expressed in logit, for the sub-groups analysis. One logit is the distance along the line of the variable that increases the odds of observing the event specified in the measurement model by a factor equal to Euler’s constant *e*. All logits are the same length with respect to this change in the odds of observing the indicative event. Higher scores represent higher perceived manual ability. **d** Slightly impaired subjects; **e** moderately impaired subjects; **f** severely impaired subjects. Green circles, yellow squares, and red stars represent PUL scores of slightly impaired, moderately impaired, and severely impaired subjects respectively. Straight lines connect data from the same participant. Grey boxes identify interquartile ranges and the black lines highlight the median values. Asterisks indicate statistical differences ($p-value < 0.05$) between groups, tested with Friedman’s and post-hoc comparisons

At baseline assessment (i.e., T0), only 9 patients achieved an entry score equal to or higher than four points and, hence, performed the shoulder items. They all belonged to the slightly impaired group. The results are shown in Fig. 5 for the whole population analysis, and in Fig. 6 for sub-groups analysis. The whole population under investigation did not show significant improvements with the passive device (Delta PUL = 1.00 [− 2.00 to 5.75], $p-value$ T0 vs T1 = 0.980). With the semi-active device, instead, patients obtained a statistically significant improvement according to Friedman’s test ($p-value$ T0 vs T1 = 0.006). However, the improvement detected by the PUL module was lower than the MDC (Delta PUL = 3.00 [0.00– 9.50]), highlighting the need to classify patients into different groups according to their residual ability. Considering slightly impaired patients, we did not observe significant changes with both devices (Delta PUL = − 1.50 [− 3.00 to 0.00] for Passive, Delta PUL = 0.00 [− 2.00 to 0.00] for Semi-Active, Friedman’s test $p-value = 0.057$). Moderately impaired patients obtained significant improvements with both arm supports (Delta PUL = 5.50 [2.50 to 6.00], $p-value = 0.046$ for Passive, Delta PUL = 5.50 [2.50 to 9.00], $p-value = 0.004$ for Semi-Active). The highest improvements were observed at the elbow level, while at the wrist and fingers level, patients obtained a slight worsening. In particular, it was mainly due to the item Q of the PUL module that involves the prono-supination of the wrist. Both devices, in fact, blocked the compensatory movement that these patients usually rely on to completely rotate their wrist. Finally, severely impaired patients showed different results with the two devices. Indeed, with the passive device, they slightly increased their total PUL score. However, this improvement was not significant (Delta PUL = 2.00 [− 4.00 to 5.75], $p-value = 0.574$) . By contrast, with the semi-active device they significantly increased their upper limb functionality (Delta PUL = 10.00 [5.00–13.75], $p-value = 0.019$). The improvement was observed at the elbow level, while at the wrist and fingers level, the device did not help patients, according to the PUL module.

### Abilhand questionnaire results

According to Abilhand results, patients improved their perceived ability to perform ADLs with both ADs, but the increase was significant only with the semi-active device (Delta Abilhand = 0.33 [− 0.33 to 1.35], $p-value = 0.165$ for Passive, Delta Abilhand = 0.82 [0.00 to 2.21], $p-value = 0.002$ for Semi-Active). Abilhand results for all patients and for the different sub-groups are shown in Fig. 6 and in Table 2.

**Table 2:** Abilhand results for all the subjects and for each patients’ group

	T0				T1 Passive				T1 Semi-Active			
Patient group	Abilhand (logit)	Easy Items	Difficult Items	Impossible Items	Abilhand (logit)	Easy Items	Difficult Items	Impossible Items	Abilhand (logit)	Easy Items	Difficult Items	Impossible Items
All	1.53 [0.85 to 3.00]	12	10	0	2.19 [1.13 to 3.32]	16	6	0	2.70 [1.74 to 4.54]	18	4	0
Slightly impaired	3.00 [1.88 to 4.17]	19	3	0	2.49 [1.99 to 5.07]	16	6	0	2.70 [2.23 to 2.85]	19	3	0
Moderately impaired	1.53 [1.52 to 2.85]	12	10	0	2.49 [1.33 to 3.75]	16	6	0	4.26 [2.35 to 4.81]	22	0	0
Severely impaired	-0.20 [-2.50 to 1.33]	3	16	3	0.94 [-1.56 to 2.72]	5	17	0	1.74 [-1.77 to 3.21]	13	9	0

In the group of slightly impaired patients, we observed a slight reduction in Abilhand results. However, this change was not significant with any device (Delta Abilhand = − 0.18 [− 0.54 to 0.24] for Passive, Delta Abilhand = 0.00 [− 0.94 to 0.49] for Semi-Active, Friedman’s test $p-value = 0.697$). Moderately impaired patients showed different results with the involved ADs. Indeed, with the passive device they did not perceive a significant increase in their upper limb ability (Delta Abilhand = 0.62 [− 0.20 to 2.22], $p-value = 0.331$). Whilst with the semi-active device participants felt a significant improvement in their manual ability (Delta Abilhand = 1.49 [0.98–2.40], $p-value = 0.001$). Finally, severely impaired patients felt a significant improvement in their ability to perform ADLs with both devices (Delta Abilhand = 0.77 [0.08–1.48], $p-value = 0.026$ for Passive, Delta Abilhand = 1.64 [0.11–2.58], $p-value = 0.009$ for Semi-Active).

### System usability scale results

Considering the SUS scale, across all patients, the SUS results were equal to 70.00 [63.13–82.50] and 80.0 [60.63–87.50] points for the passive and semi-active device, respectively. These results were respectively “good” and “excellent”. Also, slightly impaired patients evaluated the semi-active device as “excellent” (82.50 points, [62.50–87.50]) and the passive device as “good” (73.75 points, [66.25–83.75]). The same classification was maintained in the moderately impaired group (Semi-Active 82.5 [67.50–87.50] points, Passive 72.5 [56.25–81.25] points). Severely impaired patients judged the usability of the semi-active device as “excellent” (77.5 points, [60.00–80]). By contrast, these patients evaluated the passive device with “almost-good” usability (67.50 points, [65.00–83.13]). This score was slightly below the average threshold defined by Sauro (i.e., 68 points) [34].

### Qualitative results

**Fig. 7:**
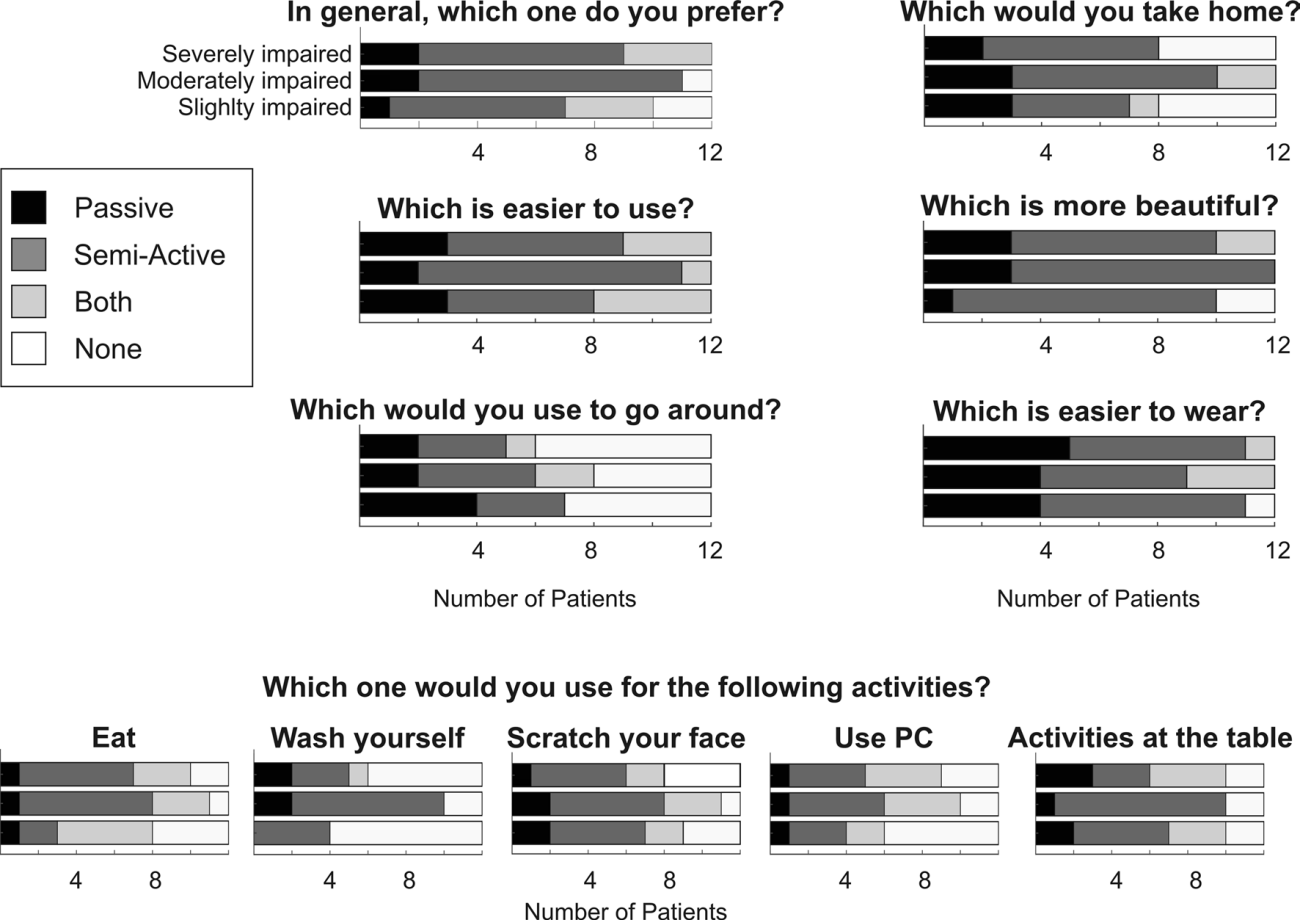
Responses to the final satisfaction questionnaire proposed to patients. Each row reports the number of responses for the three groups of patients (i.e., slightly, moderately, and severely impaired, 12 patients for each group). Black, dark grey, light grey, and white bars represent, respectively, the number of answers relative to the passive device, the semi-active device, both devices, and none

Results of the final questionnaire are reported in terms of the frequency of responses in Table 3 and Fig. 7. We observed that, in general, the semi-active was preferred to the passive device (22 patients). Most of the participants reported that some ADLs were easier with the ADs. Indeed, they were able to complete a range of ADLs involving the upper limbs faster, more independently, and with reduced compensatory movements. Eating, touching the face, and playing activities at the table were most often positively impacted by the semi-active device. In particular, patients felt an improved ability to eat and drink without the assistance of their caregivers, and to scratch their face, comb hair, or wear the glasses alone. During these actions, they felt increased self-esteem. For personal hygiene activities, 15 patients, mostly moderately impaired, would prefer the semi-active device, whereas 16 participants would not use any device.

**Table 3:** Final satisfaction questionnaire proposed to patients

**Questions**	**Passive**	**Semi-Active**	**Both**	**None**
1. In general, did you prefer to use the passive, semi-active, both devices or none?	5 (1/2/2)	22 (6/9/7)	6 (3/0/3)	3 (2/1/0)
Which is the best device to use for the following activities?				
2. Eat	3 (1/1/1)	15 (2/7/6)	11 (5/3/3)	7 (4/1/2)
3. Wash yourself	4 (0/2/2)	15 (4/8/3)	1 (0/0/1)	16 (8/2/6)
4. Scratch/clean your face	5 (2/2/1)	16 (5/6/5)	7 (2/3/2)	8 (3/1/4)
5. Use PC, PlayStation or TV remote control	3 (1/1/1)	12 (3/5/4)	10 (2/4/4)	11 (6/2/3)
6. Play activities at the table	6 (2/1/3)	17 (5/9/3)	7 (3/0/4)	6 (2/2/2)
7. Which is easier to use?	8 (3/2/3)	20 (5/9/6)	8 (4/1/3)	0
8. Which is more beautiful (in terms of design)?	7 (1/3/3)	25 (9/9/7)	2 (0/0/2)	2 (2/0/0)
9. Which device Would you prefer to go around on the wheelchair?	8 (4/2/2)	10 (3/4/3)	3 (0/2/1)	15 (5/4/6)
10. Which is easier to assembly and wear?	13 (4/4/5)	18 (7/5/6)	4 (0/3/1)	1 (1/0/0)
11. If you could choose, which device would you take home?	8 (3/3/2)	17 (4/7/6)	3 (1/2/0)	8 (4/0/4)

Total number of responses and by group (slightly/moderately/severely impaired patients)

For subjects with low muscular weakness often the ADs restricted the range of motion and their ability to lean forward to reach objects. By contrast, severely impaired participants reported that, given their high level of disability, they preferred to be helped directly by caregivers. Patients expressed greater variability regarding the effectiveness of both devices to use the PC or Play Station. Indeed, 12 patients preferred the semi-active, 10 appreciated equally both devices, 3 preferred the passive device, whereas 11 would not choose any device. In particular, most of the severely impaired patients reported that both devices negatively impacted their ability to use the PC and type on the keyboard. Moreover, the passive device increased the effort required to lower patients’ arm against the antigravity force. Participants agreed about the ease of use of both ADs. 20 patients preferred the semi-active device, and 8 patients preferred the passive one. We obtained similar results also considering the arm supports’ design and ease of assembly. Patients generally appreciated the aesthetic aspect of ADs and found the assembly and wearing procedures comfortable. Also in this case, the semi-active device received more preferences (25 for aesthetic aspect, 18 for ease of assembly). 15 participants would go around without any AD mounted on the wheelchair. Finally, most patients (17) reported that they would keep in their home the semi-active, 7 the passive, 2 both devices, and 7 none of them. Therefore, this questionnaire showed that the semi-active device achieved better results in terms of performance and users’ satisfaction.

## Discussion

We conducted a rigorous RCT that aimed to evaluate externally-assessed functional improvements, self-perceived improvements, and usability of two commercial upper limb ADs in people with MD. This study highlighted that the effectiveness of upper limb supports depends on the level of impairment. Indeed, the degenerative nature of MD changes the way the device is used and is beneficial for patients. Our results are consistent with existing evidence of the need for a person-centered approach to ADs assessment, and prescription for people with MD [16].

In USEFUL project, we divided patients according to their muscular strength at the deltoid level, detected through the MRC index.

### Discussion of functional improvement in light of different residual ability levels

Slightly impaired patients (i.e., $2.5 < MRC \le 4$) did not show significant clinical changes with the use of both devices. They mainly perceived they had the same upper limb abilities with and without ADs. Some patients reported that the arm supports limited their achievable volume of work. In particular, the passive device prevented them from extending the trunk and, hence, they felt uncomfortable when they had to reach distant points. Anyway, some of them found these ADs beneficial for activities requiring arm lifting (e.g., combing hair, shaving) or move weights. A commonly reported comment after the tests was that, thanks to arm support, they reduced arm fatigue and improved endurance.

The moderately impaired group (i.e., $1 < MRC \le 2.5$), instead, obtained significant improvements with both ADs, reducing compensatory movements, as detected by the PUL module. They reported improved upper limb functions with the use of both ADs. Indeed, with the Abilhand questionnaire, patients reported that more actions were easier with the arm supports than unassisted. However, the improvements found in the Abilhand questionnaire were statistically significant only with the semi-active device. Also in the final questionnaire, most of the moderately impaired patients reported greater preferences for the semi-active device, justifying the preference since they felt limited by the passive one when performing desk activities (e.g., writing, typing on the keyboard). This suggests that a semi-active device could be more beneficial for patients characterized by a moderately reduced arm strength.

Finally, with severely impaired patients (i.e., $MRC \le 1$) the two devices showed different effectiveness. Indeed, the clinical benefit underlined by the PUL module was high and significant only with the semi-active device. However, these subjects perceived more actions to be easier to be done with both devices. Nevertheless, with the semi-active one, they could reach a higher level of upper limb ability, as detected by both PUL and Abilhand. With the passive device, instead, they still remained strongly limited during most of ADLs. The greater improvements induced by the semi-active device were identified from both quantitative and qualitative outcome measures.

With the semi-active device, moderately and severely impaired patients strongly relied on the possibility to change the level of antigravity compensation with the remote control, and this feature was beneficial. They exploited it especially when they had to lift their arm (e.g., reaching the mouth). At the same time, they took advantage of it to reduce the antigravity compensation for tasks at the table level (e.g., writing), according to their residual ability and arm’s weight. By contrast, with the passive device users were not able to autonomously modify the number of elastic bands that carried out the antigravity compensation set during the mounting phase. This feature proved to be a limit, especially for patients with high arm weakness. Indeed, in some cases the passive device did not offer enough compensation to completely lift the arm. At the same time, the user had to increase the effort to lower the arm against AD’s resistance. It suggests that moderately and severely impaired patients have inadequate proximal upper limb strength for an effective use of passive ADs. These results are consistent with other studies, that reported the need for sufficient upper limb strength to overcome the resistance of the AD to lower the arm and effectively use the device [8, 14, 16].

Severely impaired patients acquired more independence during several ADLs thanks to arm supports. However, with passive and semi-active devices they still remained unable to perform some tasks (e.g., personal hygiene). During other activities, instead, they felt limited by the devices. In particular, the inability to stabilize the forearm on a table surface or wheelchair armrest while using the AD was an issue for some participants and impacted negatively in some ADLs (e.g., typing on the keyboard, using a laptop). According to the results of this study, severely impaired patients benefit more from the semi-active device, given the difficulty to autonomously change the working plane. From the results on the group of severely impaired patients and with informal feedback from participants, we can suggest that these subjects could benefit more from a fully-active device, that drives their arm through the 3D space, supporting both the antigravity and the planar movements [35].

While only 6 participants increased their PUL score above the set MCID threshold (i.e., 12 points), the actual benefit of these devices does not depend on their clinical relevance alone. In fact, the functional benefit and the increased ability in performing ADLs were relevant for patients even when not reaching the clinical relevance for the PUL value.

We noticed that the improvement, according to both the externally-assessed and self-reported scales, was more correlated with the level of residual muscle strength, rather than the specific MD type. In fact, patients diagnosed with different types of MD have diverse progressions of muscular and functional loss, due to the specific time course of the pathology. We observed that participants with Congenital and Becker MD mainly constituted the slightly impaired group. Moderately and severely impaired groups, instead, were primarily formed by people diagnosed with Duchenne or Limb-Girdle MD. However, the participants recruited, even if diagnosed with the same MD type, spanned different ages (e.g., from 12 to 24 years old for DMD). Therefore, we decided to perform the stratification for residual muscular capabilities rather than for pathology.

### Devices usability and acceptance

In general, participants found both devices easy to use and they would suggest their use. The greater improvements obtained with the semi-active one were reflected also in the SUS scale. Indeed, it obtained higher scores in all sub-groups of patients and users felt more comfortable while using it. Moreover, it allowed participants to move their trunk if they were still able to do it. In this way, they could explore a wider range of motion. The main drawback identified by patients was the need to use an electric wheelchair. Otherwise, it requires to be connected to the power line, and the usability was limited to a fixed position. Considering the passive device, instead, participants highlighted its lightness and reduced encumbrance, because it did not exceed the wheelchair volume. Nevertheless, movements were perceived as less smooth and fluent. Moreover, it blocked the movements, often useful and compensatory, of the trunk.

A large number of participants would not use the device out of their homes. It could mean that patients prefer this kind of device for daily home activities (e.g., eating, drinking, combing hair) rather than having it mounted on the wheelchair throughout the day. The results obtained in this study are consistent with those obtained in other feasibility studies conducted on people with MD, which underlined a general increase in externally-assessed measures [14, 15, 18] and self-perceived scales [1], depending on patients’ ability.

This RCT demonstrated that ADs can help people with MD during their daily life. As a consequence, the functional benefits detected by the clinical scales could be translated into improvement of the quality of life. It has provided some considerations for future researches in the field of assistive technologies for people with MD and other neuromuscular diseases.

It has to be noted that the training period with each AD was limited to only three days. In a preliminary phase of the study, for a limited number of patients, we extended the training period. Patients could bring both devices to their homes for a period of 15 days. However, we noticed that the longer training period did not influence the performances in both externally-assessed and self-perceived evaluations. This highlighted the fact that both devices are very user-friendly and that only a few hours of training are needed for the user to effectively control the exoskeleton and to leverage the assistance provided.

### Study limitations

Our study considered two illustrative ADs, Wrex and Ayura, as representative of the respective AD category: passive and semi-active devices. This is undoubtedly a limitation of the results obtained in the crossover RCT, since some specific design and functional characteristics are peculiar to Wrex and Ayura, and cannot be extended to other devices of the same category. However, we believe that the kind of assistance provided by the two systems is a characteristic shared by all devices belonging to the same category. Specifically, passive systems are characterized by providing a stationary level of assistance, that cannot be tuned continuously (e.g., with the addition/removal of elastic bands for the Wrex, or with the adjustment of spring tensions). On the other hand, with semi-active or fully active devices, the user can modulate the amount of assistance in real-time, usually with buttons or other types of controllers (e.g., voice recognition, joysticks, etc.). In general, motorized systems can generate higher forces, therefore being able to assist users suffering from higher muscular weakness. Moreover, the usefulness of both passive and semi-active devices may be limited by joint contractures that are often present in people with MD [36, 37]. Contractures can, indeed, reduce the joint range of motion, and thus the exploration of the space, even in presence of a support.

As a remark, we would like to highlight that we have in this paper investigated the functional improvement at the upper limb level. However, when coming to effective use in daily life, this is not the only element to be considered. Indeed, despite all the developmental efforts, few devices are nowadays commercially available. Only a small portion of the people who can potentially benefit from the use of arm support actually uses one [38]. In fact, these devices are expensive, and they could create additional costs in patients’ usual care. The absence of extensive validation has prevented the health care systems to recognize these devices in the accreditation lists, limiting the accessibility to the end-users. Moreover, considering active and semi-active devices, they have an higher cost, their use depends on electricity, with relevant consequences in usability in daily life.

### Future sights

Future rigorous RCT could focus on different neuromuscular diseases (e.g., multiple sclerosis, spinal muscular atrophy) to characterize the impact of ADs on these patients. Moreover, further research is needed to understand the level of strength required to effectively use a passive AD. This information is necessary to guide the prescription of ADs and funding decisions. In addition, proper testing of these assistive devices in daily life environment, which would provide evidence on the real effectiveness and usability of mobile arm supports, might be investigated through the use of a Technology Acceptance Model [39].

Fatigue and impaired upper limb function are commonly identified by individuals with MD as problematic symptoms and are known to negatively impact independence and quality of life [13, 16]. To deepen this aspect, future studies should include outcome measures related to fatigue and endurance, both to investigate self-perceived fatigue (e.g., Borg scale) or externally assessed muscular fatigue (e.g., electromyography measurements). In fact, upper limb ADs may reduce the impact of these symptoms and, hence, improve participation and independence in ADLs.

As further investigation directions, a longitudinal study spanning several weeks or months could study the effect of ADs as training tools to slow down the loss of motor functions that affects people with neuromuscular disorders. In fact, a long term daily use of an exoskeleton could provide additional benefits, going beyond the immediate improvement of functional movements. For instance, the muscular tone could be preserved, since the user is more prone to move his/her upper limbs, being facilitated by the assistance provided by the device. A longitudinal study could also provide evidence about the relationship between the use of ADs and the consequent delays of fatigue onset and reduced fatigue [40].

In this study, we have investigated monolateral support. Further studies might test the use of bilateral support to perform bilateral tasks, such as lifting a heavier object or buttoning a shirt. Beside the possible functional improvement, however, we should consider double cost and encumbrance, and the fact that most impaired patients use one hand to control the wheelchair joystick.

Finally, an important direction of development is related to more natural and immediate control of the AD by the subject. For example, with the semi-active device, the assistance can be modulated by the user, but the human-machine interface is based on very simple push-buttons, without any integrated intelligence and control of the system. Advancing the development of exoskeletons towards solutions able to respond to the patient’s need, interpreting the intention and the situation in which the subject acts, is an essential frontier to improve the effectiveness and the acceptability of AD.

## Conclusion

Muscular dystrophy is a degenerative disorder that impacts life span, as well as participation outcomes and quality of life [16, 41]. The use of assistive devices has the potential to improve the quality of life for people with MD, by enabling them to continue performing daily activities and participating in social life. In recent decades, some arm supports that aim to compensate for the loss of arm function in people with muscular weakness have been developed [38]. However, extensive validation of these kinds of devices is still missing [25].

USEFUL project investigated the effect of two commercial arms supports on the upper limb functions of people suffering from MD, following the RCT methodological approach. Most of the patients reported improved upper limb function with the use of both passive and semi-active devices. In particular, eating and drinking were the most frequently and positively activities impacted by ADs use. However, the effectiveness of each AD was related to the level of residual ability of the end-user. Slightly impaired patients maintained the same independence without and with ADs. Thanks to ADs they reduced muscular fatigue and improved endurance. Moderately impaired patients enhanced their upper limb functionality with both passive and semi-active devices. The semi-active one obtained slightly higher improvements. Finally, severely impaired subjects benefited more from the semi-active device. Indeed, consistently with other studies, inadequate strength was recognized as a barrier to passive ADs, and highlighted the need for fully active devices.

This research provides further evidence of the need to improve funding efficiencies and timeliness of ADs supply for people with neuromuscular disabilities, such as MD. Future researches are needed to improve the evidence on the effect of ADs on quality of life and diseases’ progression in subjects with degenerative disorders.

## Supplementary Information

**Additional file 1: Table S1.** Summary of statistical tests performed, relative sample size, correction for multiple comparisons and resulting p-values. NS = Non-significant (p > 0.05); THDS = Tukey's Honestly Significant Difference correction for multiple comparisons.

## Data Availability

The datasets generated and analysed during the current study are available in the Harvard Dataverse repository, together with a MATLAB live script that generates all the figures and statistical analyses reported in this work. https://doi.org/10.7910/DVN/PLH43R.
