# Supplementary material for: User-centred assistive SystEm for arm Functions in neUromuscuLar subjects (USEFUL): a randomized controlled study
Source: J Neuroeng Rehabil. 2021 Jan 6;18:4. doi: 10.1186/s12984-020-00794-z (PMC7789525; doi:10.1186/s12984-020-00794-z)
Supplement: Supplementary file 1 — Additional file 1: Table S1. Summary of statistical tests performed, relative sample size, correction for multiple comparisons and resulting p-values. NS = Non-significant (p > 0.05); THDS = Tukey's Honestly Significant Difference correction for multiple comparisons. [file 12984_2020_794_MOESM1_ESM.pdf]

Supplementary Table 1. Summary of statistical tests performed, relative sample size, correction for multiple comparisons and resulting p-values. NS = Non-significant (p > 0.05); THDS = Tukey's Honestly Significant Difference correction for multiple comparisons.

Comparison	Statistical test	Sample Size	Correction	Test results
**Total PUL Values**	*Friedman Test*<br>T0 vs T1 Passive vs T1 Semi-Active	All complete subjects (N=34)		p = 0.00280
	Post-hoc comparisons			
	T0 vs T1 Passive		THSD	NS, p = 0.98
	T0 vs T1 Semi-Active		THSD	p = 0.006
	T1 Passive vs T1 Semi-Active		THSD	p = 0.011
	Friedman Test<br>T0 vs T1 Passive vs T1 Semi-Active	Slightly impaired (N=12)		NS, p = 0.057
	Friedman Test<br>T0 vs T1 Passive vs T1 Semi-Active	Moderately Impaired (N=12)		p = 0.004
	Post-hoc comparisons			
	T0 vs T1 Passive		THSD	p = 0.0039
	T0 vs T1 Semi-Active		THSD	p = 0.046
	T1 Passive vs T1 Semi-Active		THSD	NS, p = 0.69
	Friedman Test<br>T0 vs T1 Passive vs T1 Semi-Active	Severely Impaired (N=10)		p = 0.0082
	Post-hoc comparisons			
	T0 vs T1 Passive		THSD	NS, p = 1.0
	T0 vs T1 Semi-Active		THSD	p = 0.019
	T1 Passive vs T1 Semi-Active		THSD	p = 0.019
**Delta PUL Values**	*Friedman Test*<br>T1 Passive - T0 vs T1 Semi-Active -T0	All complete subjects (N=34)		p = 0.001
**Abilhand Values**	*Friedman Test*<br>T0 vs T1 Passive vs T1 Semi-Active	All complete subjects (N=34)		p = 0.00284
	Post-hoc comparisons			
	T0 vs T1 Passive		THSD	NS, p = 0.16
			THSD	p = 0.0018

	T0 vs T1 Semi-Active<br><br>T1 Passive vs T1 Semi-Active		THSD	NS, p = 0.24
	Friedman Test<br>T0 vs T1 Passive vs T1 Semi-Active	Slightly impaired (N=12)		NS, p = 0.69
	Friedman Test<br>T0 vs T1 Passive vs T1 Semi-Active	Moderately Impaired (N=12)		p = 0.0021
	Post-hoc comparisons<br>T0 vs T1 Passive<br><br>T0 vs T1 Semi-Active<br><br>T1 Passive vs T1 Semi-Active		 THSD<br><br>THSD<br><br>THSD	 NS, p = 0.33<br><br>p = 0.0014<br><br>NS, p = 0.09
	Friedman Test<br>T0 vs T1 Passive vs T1 Semi-Active	Severely Impaired (N=10)		p = 0.0056
	Post-hoc comparisons<br>T0 vs T1 Passive<br><br>T0 vs T1 Semi-Active<br><br>T1 Passive vs T1 Semi-Active		 THSD<br><br>THSD<br><br>THSD	 p = 0.026<br><br>p = 0.0088<br><br>NS, p = 0.93
